# Effects of the Addition of Iron and Chromium on the Structure and Properties of the Ni-Co-Mn-In Alloy

**DOI:** 10.3390/ma18194597

**Published:** 2025-10-03

**Authors:** Edyta Matyja, Krystian Prusik

**Affiliations:** Institute of Materials Engineering, Faculty of Science and Technology, University of Silesia in Katowice, 75 Pułku Piechoty 1a, 41500 Chorzów, Poland; krystian.prusik@us.edu.pl

**Keywords:** magnetic shape memory alloys, microstructure, Fe-doped, Cr-doped, martensitic transformation

## Abstract

In this work, small amounts of Fe or Cr were added to Ni_47_Co_3_Mn_36.5_In_13.5_ alloy (x = 0) to produce five-component alloys with nominal compositions of Ni_47_Co_3_Mn_35.5_In_13.5_Fe_1_, Ni_47_Co_3_Mn_33.5_In_13.5_Fe_3_, Ni_47_Co_3_Mn_35.5_In_13.5_Cr_1_, and Ni_47_Co_3_Mn_33.5_In_13.5_Cr_3_, which are denoted as Ni_47_Co_3_Mn_36.5−x_In_13.5_Fe_x_/Cr_x_ (x = 1, 3 at.% Cr/Fe) series or as Mn-series (due to the addition of alloying elements instead of Mn), and Ni_47_Co_3_Mn_36.5_In_12.5_Fe_1_, Ni_47_Co_3_Mn_36.5_In_10.5_Fe_3_, Ni_47_Co_3_Mn_36.5_In_12.5_Cr_1_, and Ni_47_Co_3_Mn_36.5_In_10.5_Cr_3_, denoted as Ni_47_Co_3_Mn_36.5_In_13.5−x_ (x = 1, 3 at.% Cr/Fe) series or In-series (due to the addition of alloying elements instead of In). The polycrystalline alloys were produced using the vacuum arc melting technique. The as-received alloys were characterized in structure, homogeneity, phase composition, martensitic transformation, and microhardness. The results showed that the addition of 1 at.% of Cr or Fe positively impacted the microstructure of the alloys. The quaternary alloy exhibited a single-phase coarse-grained structure. The addition of Fe and Cr (1 at.%) caused microstructure refinement with small Fe/Cr- and Co-rich γ particles appropriately distributed in the matrix, while the addition of 3% Fe or Cr resulted in γ formation in a dendritic form distributed more randomly. The addition of 1 at.% and 3 at.% of Cr or Fe significantly influenced the martensitic transformation temperatures. The microhardness increased by 21% in the Ni_47_Co_3_Mn_33.5_In_13.5_Fe_3_ alloy compared to the quaternary alloy.

## 1. Introduction

NiMn-based ferromagnetic shape memory alloys (FSMAs) have attracted interest in recent years due to their high potential for application as magneto-mechanical actuators, sensors, or energy harvesting devices. The Ni-Mn-Z (Z = Ga, Sn, In) alloys exhibit multifunctional properties such as high recoverable strain, giant magnetocaloric effects, and elastocaloric and barocaloric effects. These alloys undergo reversible martensitic transformation (MT), which can be controlled by the temperature and an external magnetic field or stress related to the reversible martensitic transformation (MT) [1,2,3,4,5].

The giant inverse magnetocaloric effect (MCE) near room temperature was observed in off-stoichiometric Ni-Mn-Z (Z = Ga, Sn, In) alloys. The inverse MCE related to the first-order martensitic transformation makes them promising for refrigeration applications with environmentally friendly cooling technologies [6,7,8,9,10].

However, despite their interesting properties, the practical use of FSMAs is hindered by their intrinsic brittleness, low ductility, and weak mechanical properties. The brittleness originates from their intermetallic structure and weak cohesive grain boundary brittleness, resulting in intergranular fracture behavior [11,12,13]. The microstructural modifications of FSMAs may improve their mechanical properties to overcome the limitations mentioned earlier.

Elemental doping is one of the effective modification routes to tailor the structural and magnetic properties of FSMAs. The substitution of transition metals such as Fe, Cr, and Co into Ni-Mn-based alloys has been explored to improve ductility, enhance cyclic stability, and optimize magnetocaloric and elastocaloric effects [2,14,15,16,17,18,19]. Adding a fourth element causes the formation of precipitates at the grain boundaries in FSMAs, which enhances their strength and ductility.

Pfeuffer et al. [14] showed that the precipitated Fe-rich γ phase nucleated at the grain boundaries, hindering intergranular fracture during cyclic loading. Therefore, a large elastocaloric effect of −4.5 K was observed in 16000 cycles without degradation. Feng et al. [20] observed an improvement in mechanical properties in Ni-Mn-In alloy with Fe addition. This resulted from the second-phase precipitation and a change of fracture type from intergranular cracks to transgranular cleavage cracks with the increase in Fe content. Similar conclusions were presented by Tan et al. [21], who observed an increase in maximum compressive strength and strain in a Ni_47_Fe_3_Mn_38_Sn_12_ alloy. They also observed an increase in compressive strength from 215.0 to 725.4 MPa and in compressive strain from 4.9% to 9.3%, with an increase in Fe content from 0 at.% to 4 at.% [22]. Additionally, they proved that Fe atom substitution enhances the ferromagnetism of Ni_50_Mn_39_Sn_11_ alloy. Halder et al. [23] studied the effect of Fe substitution on the magnetic properties of Ni-Mn-In Heusler alloys. They proved that various substitutions at Ni or Mn sites can tailor the structural and magnetic properties. The change in magnetic entropy was around 5.5 J kg^−1^ K^−1^ for Ni_48_Fe_2_Mn_35_In_15_ and 4.5 J kg^−1^ K^−1^ for Ni_50_Mn_34_FeIn_15_. Li et al. [24] observed grain refinement in Ni-Mn-In alloy when the Fe content increased up to 2 at.%.

Large magnetic entropy changes and a magnetic field-induced phase transformation were obtained in Ni_50_Mn_34_In_14_Fe_2_ alloys, which makes this alloy attractive for use in practical multifunctional devices [25]. The maximum effective refrigeration capacity of a Mn_49_Fe_1_Ni_41_In_9_ ribbon was 137.1 J kg^−1^ under a magnetic field of 30 kOe. Zhang et al. [26] showed that magnetostructural coupling, in which martensitic transformation occurs from ferromagnetic austenite to the paramagnetic martensite, occurred in Ni_50−x_Fe_x_Mn_38_Sn_12_ alloys with Fe contents of x = 0.5–4.2. Furthermore, the addition of 4.2 at.% of Fe enhanced the magnetic properties because it increased the difference between the magnetization of austenite and martensite, ΔM, which was 58 emu/g under 50 kOe. Tan et al. [22] proved that the enhancement in magnetic-field-induced martensitic transformation by Fe doping originated from tuning antiferromagnetic austenite to a ferromagnetic state by substituting Fe for Ni.

Akkera et al. [27] studied the effect of Cr addition on the structure, martensitic transformation, and mechanical properties of Ni-Mn-In thin films. They showed that an increase in Cr content leads to a decrease in grain size, an increase in martensitic transformation temperatures, and an increase in hardness. They also found [28] in the Ni_51.1_Mn_34.9_In_9.5_Cr_4.5_ film a magnetic entropy change of ΔS_M_ = 7.0 mJ cm^−3^ K^−1^ at 302 K and a refrigerant capacity of 39.2 mJ cm^−3^ under an applied field of 2 T, which indicates that the Cr-doped Ni-Mn-In alloy thin films are potential candidates for room-temperature micro-length-scale magnetic refrigeration applications. Pandey et al. [29] found that substituting a small amount of Cr on the Ni site decreases martensitic and Curie temperatures. Additionally, the temperatures are sensitive to pressure. The largest value of refrigeration capacity RC = 340 J kg^−1^ (ΔH = 5 T) was observed in Ni_48_Cr_2_Mn_37_In_13_ alloy, making this alloy a promising material for application in magnetocaloric refrigeration technology.

Although the impact of Fe [14,20,21,22,23,24,25,26] and Cr [27,28,29] additions on ternary Ni-Mn-In alloys has been studied, their effect on quaternary Ni-Co-Mn-In alloys remains unknown. The present study aimed to investigate the effect of Fe and Cr additions on the structure and properties of Ni-Co-Mn-In-(Fe/Cr). The alloy compositions were tuned based on the electron concentration ratio (e/a) to obtain martensitic transformation near room temperature.

## 2. Materials and Methods

**Figure 1:**
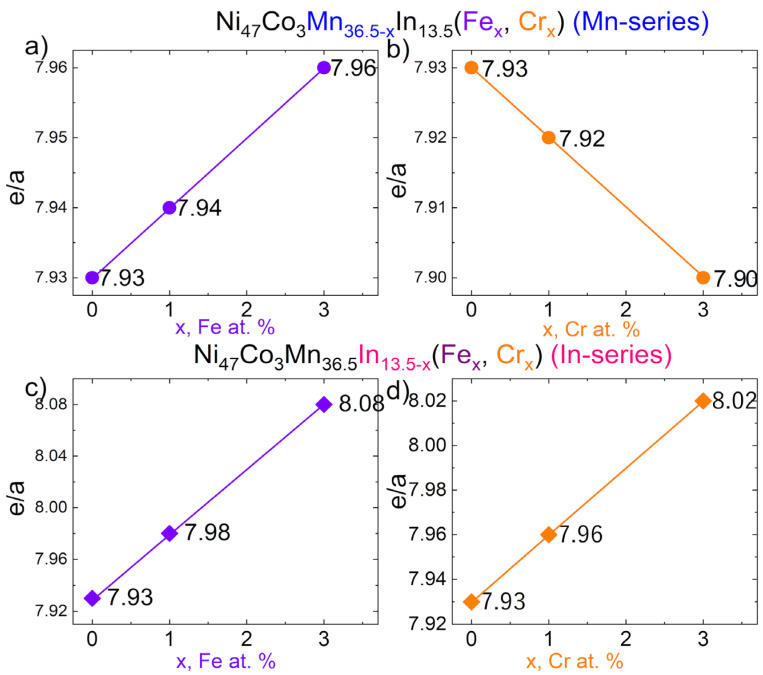
The theoretical electron concentration ratio (e/a) for Ni_47_Co_3_Mn_36.5−x_In_13.5_ (Mn-series) with x = 0, 1, 3 at.% of Fe (**a**) and Cr (**b**) and for Ni_47_Co_3_Mn_36.5_In_13.5−x_ (In-series) with x = 0, 1, 3 at.% of Fe (**c**) and Cr (**d**).

Elemental powders of Ni (99.9%; 46 µm), Co (99.9%; 0.5–1.5 µm), Mn (99.5%; 44 µm), and In (99.99%; 44 µm), supplied by Strem Chemicals, and Cr (99%; APS < 150 µm) and Fe (99%; APS < 60 µm), delivered by KAMB Import-Export, were precisely weighted to obtain samples with nominal compositions Ni_47_Co_3_Mn_36.5−x_In_13.5_ (x = 1.3 at.% Cr/Fe and Cr, Fe) and Ni_47_Co_3_Mn_36.5_In_13.5−x_ (x = 1.3 at.% Cr/Fe) (Table 1, Figure 1). As-prepared powders were mixed using a Fritsch Pulverisette 6 planetary ball mill to homogenize the powder mixture. Weight loss after mixing was less than about 1 wt.%. Then, powders were cold-pressed using a hydraulic press, and the obtained green compacts were used to produce bulk alloys by vacuum arc melting (VAR) using the protective atmosphere (Ar). Each sample was prepared under the same conditions and remelted five times to promote homogeneity. The as-received buttons were cut by an Electric Discharge Machine (EDM) using low current. Longitudinal and cross-sectional metallographic specimens were prepared by mechanical grinding and polishing.

X-ray diffraction (XRD) investigations were carried out using the Panalytical Empyrean diffractometer (Malvern Instruments, Malvern, UK) with the Cu anode (wavelength of Cu k_α_ = 1.54056 Å) working at an electric current of 30 mA and 40 kV voltage. The diffractometer was equipped with a PIXcell^3D^ detector (Malvern Instruments, Malvern, UK). The cross-sectional specimens after grinding were used for XRD measurements.

The microstructure was observed using a Scanning Electron Microscope JEOL JSM-6480 in backscattered electron (BSE) mode with an accelerating voltage of 20 kV. Chemical composition was measured using Energy-Dispersive X-ray spectroscopy (IXRF, Oxford Instruments), with which the SEM is equipped. The crystallographic orientation image maps (OIMs) were recorded using the electron backscatter diffraction (EBSD) technique (HKL system equipped with NordlysII camera). The measurements were carried out using an accelerating voltage of 20 kV and a working distance (WD) of 20 mm. The microstructure observations at the nanoscale were carried out using a Transmission Electron Microscope (TEM) JEOL JEM-3010, operating at 300 kV. The TEM specimens were cut using an EDM, followed by mechanical grinding and electropolishing in a solution of perchloric acid dissolved in methanol and cooled to 0 °C.

The phase transformations were examined using Differential Scanning Calorimetry (DSC), Mettler Toledo, in a temperature range from −120 to 250 °C with a cooling/heating rate of 10 °C/min. The microhardness was measured using a MicroVickers tester 401MVD with a load of 5 kG and a loading time of 10 s.

## 3. Results and Discussion

### 3.1. X-Ray Diffraction (XRD)

**Figure 2:**
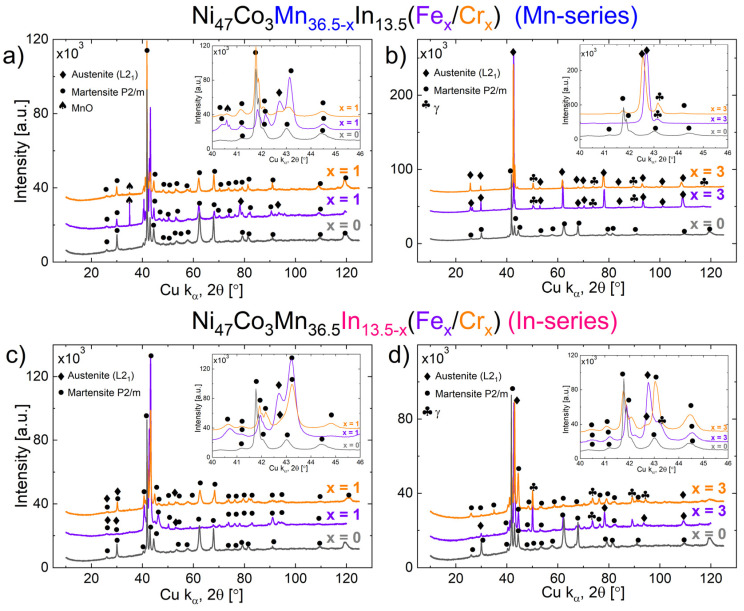
X-ray diffraction patterns for Ni_47_Co_3_Mn_36.5−x_In_13.5_ (denoted Mn-series) for x = 0 and 1 at.% Cr/Fe (**a**) and x = 0 and 3 at.% Cr/Fe (**b**), and for Ni_47_Co_3_Mn_36.5_In_13.5−x_ (denoted In- series) for x = 0, 1 at.% Cr/Fe (**c**) and x = 0 and 3 at.% Cr/Fe (**d**).

Figure 2 shows the XRD patterns recorded for Ni_47_Co_3_Mn_36.5−x_In_13.5_ (denoted Mn-series) for x = 0 and 1 at.% Cr/Fe (Figure 2a) and x = 0 and 3 at.% Cr/Fe (Figure 2b), and for Ni_47_Co_3_Mn_36.5_In_13.5−x_ (denoted In-series) for x = 0 and 1 at.% Cr/Fe (Figure 2c) and x = 0 and 3 at.% Cr/Fe (Figure 2d).

The quaternary alloy Ni_47_Co_3_Mn_36.5_In_13.5_ (x = 0) exhibited a single-phase monoclinic martensite structure characterized by the P2/m space group. The determination of its phase was based on the analysis of a non-modulated martensite structure (2M) with lattice parameters a_0_ = 4.405 Å, b_0_ = 5.553 Å, and c_0_ = 12.95 Å, as referenced by ICDD 04-017-3752 for the Ni_46_Mn_41_In_13_ composition. In further studies, the presence of martensite, mainly 14M, was confirmed using the EBSD technique.

As one can see, the addition of 1 at.% of Cr or Fe to the Ni_47_Co_3_Mn_36.5_In_13.5_ alloy (both in the Mn-series and the In-series—Figure 2a,c) resulted in the appearance of the austenite phase. The austenite phase was identified as an L2_1_ (Heusler) structure with a face-centered cubic cell and lattice parameter a_0_ = 5.982 Å ($Fm\bar{3}m$, 225 ICDD 04-002-8569). A shift in martensite peaks toward higher 2θ angles was observed in the alloys with Fe and Cr addition. This is especially clearly visible in the inset of Figure 2a,c; this shift is higher in the In-series than the Mn-series. It is correlated with lattice contraction, because the atomic radius of Cr (125 pm) and Fe (124 pm) is smaller than that of Mn (127 pm) and much smaller than that of In (167 pm).

In the alloys with 3 at.% of Cr and Fe (both in the Mn-series and the In-series), the γ-phase reflections are clearly visible in the diffraction patterns. The γ phase was identified as face-centered cubic with the lattice parameter a_0_ = 3.639 Å ($Fm\bar{3}m$, 225, ICDD 04-017-6736). Further microstructural investigations (by SEM and EBSD) also show γ particles in samples with 1 at.% of Cr or 1 at.% of Fe. However, the characteristic reflections of the γ phase are not visible on the diffraction patterns, because they overlap with the reflections of martensite and austenite. The alloys with 3 at.% of Fe and 3 at.% of Cr in the In-series and that with 3 at.% of Cr in the Mn-series exhibit a three-phase structure (austenite + martensite + γ). The alloys with 3 at.% Fe and 3 at.% Cr in the In-series, as well as that with 3 at.% Cr in the Mn-series, exhibit a three-phase structure (austenite + martensite + γ). The alloy with 3 at.% Fe in the Mn-series does not exhibit a martensitic phase, indicating that this alloy is two-phase (austenite + γ). The lack of martensitic phase reflections is particularly visible in inset of Figure 2b (difference between diffraction patterns for 3 at.% Cr and 3 at.% Fe in the 2θ range from 41.7 to 44.2°).

The phase analysis revealed only some peaks corresponding to manganese oxide ($Fm\bar{3}m$, 225, a_0_ = 4.44 Å ICDD 04-002-8161) (Figure 2a). The MnO comes from the surface oxidation of the bulk alloys, produced by vacuum arc melting from elemental powders. Before the XRD analysis, the surface of the specimens was polished; however, some amount of the MnO layer remained in the samples with 1 at.% Cr and 1 at.% Fe in the Mn-series. The observed relatively high intensity of (111)_MnO_ at 34,98° 2θ may result from a coarse-grained MnO phase, because the most intense peak of MnO theoretically occurs at 40.61°2θ (200)_MnO_, which is relatively low in the experimental XRD pattern (Figure 2a: inset).

### 3.2. Microstructure (SEM) and Chemical Composition (EDS)

**Figure 3:**
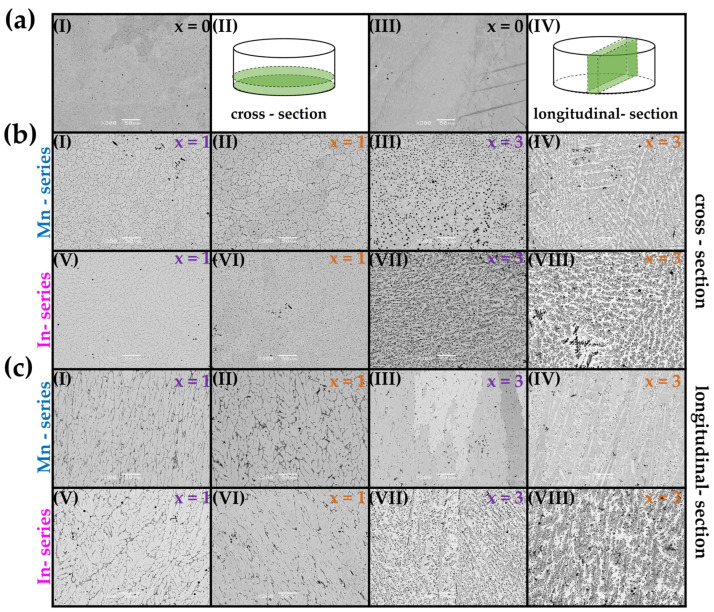
SEM BSE microstructure images of the quaternary alloy (x = 0) (**a**) observed in a cross-section (a-I) and longitudinal section (a-III) of the button, which are schematically presented in (a-II) and (a-IV), respectively. SEM BSE images of Mn-series: Ni_47_Co_3_Mn_36.5−x_In_13.5_ (x = 1 at.% Fe (I), x = 3 at.% Fe (III)—marked purple; x = 1 at.% Cr (II), x = 3 at.% Cr (IV)—marked orange); In-series: Ni_47_Co_3_Mn_36.5_In_13.5−x_ (x = 1 at.% Fe (V), x = 3 at.% Fe (VII)—marked purple; x = 1 at.% Cr (VI), x = 3 at.% Cr (VIII)—marked orange) observed in the cross-section (**b**) and longitudinal section (**c**).

Figure 3 presents the SEM BSE images (with compositional contrast) of the Ni_47_Co_3_Mn_36.5−x_In_13.5_ (x = 1, 3 at.% Cr/Fe—denoted as Mn-series) and Ni_47_Co_3_Mn_36.5_In_13.5−x_ (x = 1, 3 at.% Cr/Fe—denoted as In-series) alloys observed in the cross-section (Figure 3b) and longitudinal section (Figure 3c). The microstructure of the quaternary alloy (x = 0) is presented in Figure 3a as a cross-section (Figure 3a-I) and longitudinal section (Figure 3a-III) of the button, which are schematically presented in (Figure 3a-II) and (Figure 3a-IV), respectively. As we can see, the quaternary Ni_47_Co_3_Mn_36.5_In_13.5_ (at.%) alloy exhibits a microstructure with coarse-grained morphology in which characteristic martensitic plates are visible. The addition of 1 at.% of Fe and Cr in both series, Ni_47_Co_3_Mn_36.5−x_In_13.5_ (Mn-series) and Ni_47_Co_3_Mn_36.5_In_13.5−x_ (In-series), caused microstructure refinement, which means that the presence of small particles of γ precipitated their appearance at the grain boundaries and inside the grain, creating a subgrain-like structure in the matrix. This observed subgrain-like structure is defined by a γ-phase particle distribution rather than a crystallographic orientation, so this term is used in a morphological context. This microstructure is particularly evident in the cross-sectional specimens illustrated in Figure 3b. Furthermore, these alloys exhibit approximately round-shaped dendrites of the matrix observed in the longitudinal section. Comparing alloys with 1 at.% Fe (Figure 3b-I) and 1 at.% Cr (Figure 3b-II) contents in the Mn-series, it can be observed that Fe addition causes a greater refinement of the microstructure compared to Cr addition. In the samples with 3 at.% Fe and 3 at.% Cr contents in the In-series, a dendritic microstructure is present. An increase in the volume fraction of precipitates is observed with an increase in Fe and Cr contents from 1% to 3% and a change in their distribution. In samples with 3% Fe and 3% Cr contents, the precipitates do not create the subgrain matrix (as in the alloys with 1 at.% Fe/Cr); instead, they are distributed more randomly. It is also worth emphasizing that the round-shaped γ particles in the alloy with 3% Fe in the Mn-series are visible only in the cross-section (Figure 3b-III). In the longitudinal-section specimen of this alloy (Figure 3c-III), a difference in contrast among matrix grains is observed, which is attributed to the crystallographic orientation of large columnar grains. Subsequent investigations using the EBSD method have further confirmed this.

The EDS measurements were carried out to control the average chemical composition and determine the segregation of elements in the matrix and precipitates. Table 2 shows the average composition (EDS measurements recorded at low magnification) of Ni_47_Co_3_Mn_36.5−x_In_13.5_ (x = 0, 1, 3 at.% Cr/Fe) alloys (Mn-series) and Ni_47_Co_3_Mn_36.5_In_13.5−x_ (x = 0, 1, 3 at.% Cr/Fe) alloys (In-series) with the calculated electron concentration ratio (e/a). The e/a parameter is higher than the theoretical values. However, the trend lines are similar to the theoretical e/a dependence from at.% of Cr and Mn. This exception was noticed for the x = 3 at.% Fe in the Mn-series alloy. It should be emphasized that the quantitative analysis of EDS may be subject to error and is not as sensitive to small changes in chemical composition as the martensitic transformation temperatures (M_s_) are. Salazar-Mejía et al. [30] showed that it was difficult for the EDS measurement to detect slight differences in chemical composition. They proved that the real change in Cr substitution in one-phase Ni-Cr-Mn-In is more noticeable based on the changes in transition temperatures and magnetic properties. Therefore, the e/a calculated based on EDS values was used to verify the trend rather than absolute stoichiometry.

Another factor which may affect the calculated quantitative chemical composition is that the L line of indium is taken for quantification, whereas other elements are quantified based on K lines.

It is worth noting the weight loss during melting, due to Mn evaporation, which was controlled and kept at a similar level for all samples (about 2 wt.%).

**Figure 4:**
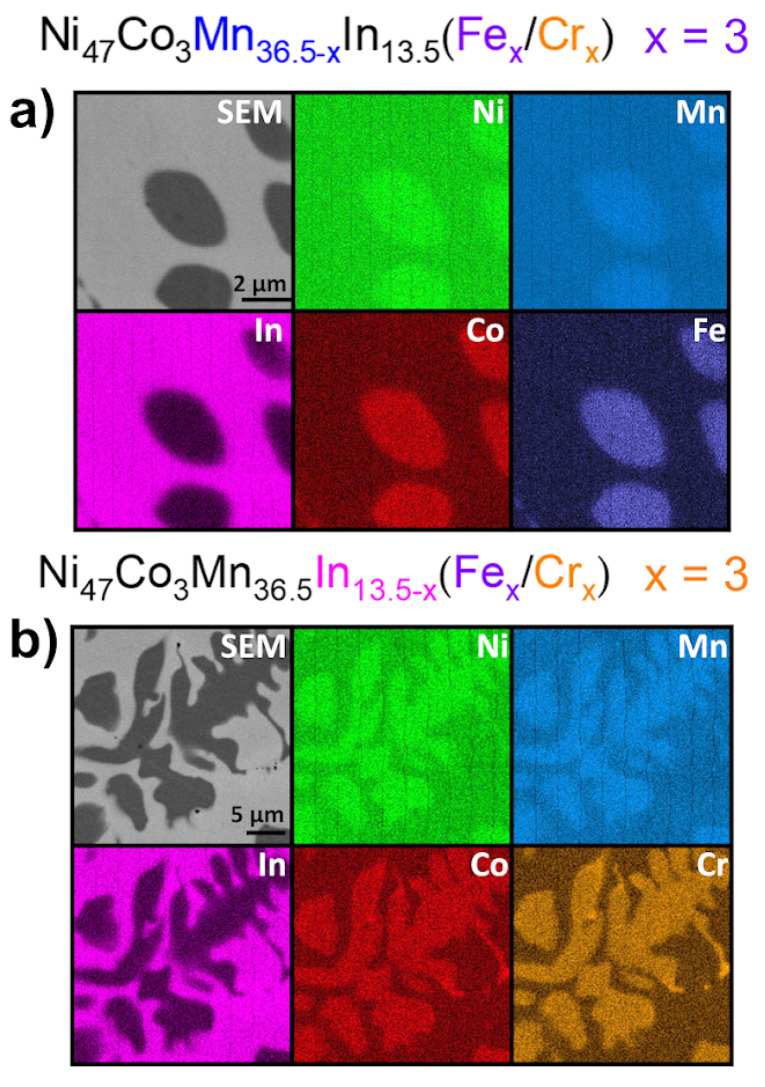
EDS chemical elemental distribution maps recorded for Ni_47_Co_3_Mn_36.5−x_In_13.5_ x = 3 at.% Fe (**a**) and Ni_47_Co_3_Mn_36.5_In_13.5−x_ x = 3 at.% Cr (**b**).

Figure 4a shows EDS elemental maps for the Ni_47_Co_3_Mn_36.5−x_In_13.5_ (x = 3 at.% Fe) alloy, and Figure 4b shows those for the Ni_47_Co_3_Mn_36.5_In_13.5−x_ (x = 3 at.% Cr) alloy. As we can see, the precipitates were enriched in Co, Ni, Mn, and Fe or Cr and depleted in In. The most significant difference in contrast occurs for the indium element, which means that In is present in the precipitates in negligible amounts (about 1–2 at.%, which was confirmed by quantitative chemical analysis). The calculated e/a ratio for precipitates is about 8.7, which means that the e/a of the matrix is lower than the theoretical value calculated based on the nominal composition.

### 3.3. Electron Backscatter Diffraction (EBSD)

**Figure 5:**
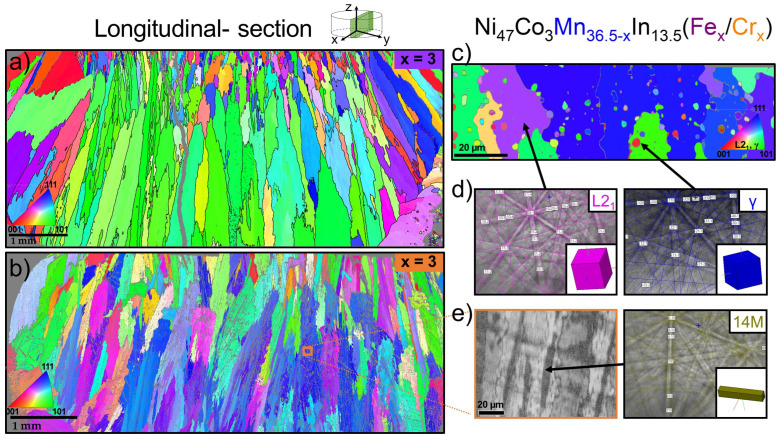
EBSD crystal orientation image maps (OIMs) from the Ni_47_Co_3_Mn_36.5−x_In_13.5_ alloy (Mn-series) with x = 3 at.% Fe (**a**) and x = 3 at.% Cr (**b**) showing the macro-longitudinal section. OIMs from the 3 at.% Fe alloy (Mn-series) recorded in a higher magnification, revealing the two-phase structure (austenite and γ particles) (**c**) with an indexed EBSD Kikuchi pattern of L2_1_ matrix and γ particles (**d**). The band contrast image of the Ni_47_Co_3_Mn_33.5_In_13.5_Cr_3_ alloy recorded in a higher magnification, revealing the martensite phase (non-indexed in macro-OIM) with the EBSD Kikuchi pattern of 14M martensite (**e**).

To confirm the phase composition and the presence of finely dispersed γ-phase particles, EBSD patterns were analyzed. Examples of EBSD Kikuchi patterns and the crystallographic orientation image maps (OIMs) projected onto the z-axis of specimens are shown in Figure 5.

The macro-OIMs recorded for longitudinal sections of the Ni_47_Co_3_Mn_33.5_In_13.5_Fe_3_ alloy (x = 3 at.% of Fe in the Mn-series) and of the Ni_47_Co_3_Mn_33.5_In_13.5_Cr_3_ (x = 3 at.% of Cr in the Mn-series) alloy are shown in Figure 5a and Figure 5b, respectively.

These OIMs revealed columnar grains oriented along the cooling direction during the arc melting process (copper plate cooling at the bottom of the sample). They confirm that the cooling rate in the produced alloys (for samples approximately 9 mm in diameter and 5 mm in height) is sufficient to form columnar grains with a specific crystallographic orientation.

The columnar grains and texture in polycrystalline shape memory alloys are favorable [31], as they may result in a higher magnetic-field-induced strain compared to randomly oriented equiaxed polycrystalline alloys. The indexed area corresponds to the matrix (austenite) phase with the L2_1_ structure. As shown in the OIM for the alloy with 3 at.% Cr (Figure 5b), some non-indexed regions (visible as gray areas) were observed. For this reason, it was not possible to determine clear grain boundaries like in the OIM for the sample with 3 at.% Fe (Figure 5a). The non-indexed areas in the macro-EBSD maps were attributed to regions containing martensite. An example of an indexed EBSD Kikuchi pattern is shown in Figure 5e, corresponding to an area recorded at higher magnification on the band contrast map. The majority of the EBSD patterns for martensite were indexed as 14M seven-layered martensite. The indexing of the martensite structure was based on matching Kikuchi lines according to the lowest MAD (Mean Angular Deviation) coefficient from the database containing martensite: 14M (P2/m, 10, a_0_ = 4.35 Å, b_0_ = 5.52 Å, c_0_ = 29.89, β = 93.2), 10M (P2/m, 10, a_0_ = 4.23 Å, b_0_ = 5.62 Å, c_0_ = 21.10, β = 89.0), body-centered tetragonal martensite (bct I4/mmm, 139, a_0_ = 2.74 Å, c_0_ = 3.28 Å, and bct a_0_ = 4.18 Å, c_0_ = 5.28 Å), and L1_0_ martensite (I4/mmm, 139 a_0_ = 3.91 Å, c_0_ = 6.71 Å).

Figure 5c shows the OIM for Ni_47_Co_3_Mn_33.5_In_13.5_Fe_3,_ revealing a two-phase microstructure consisting of matrix grains and particles. The EBSD measurements confirmed the presence of austenite with an L2_1_ structure (matrix) and γ particles. Examples of indexed EBSD Kikuchi patterns are presented in Figure 5d.

### 3.4. Transmission Electron Microscopy (TEM)

**Figure 6:**
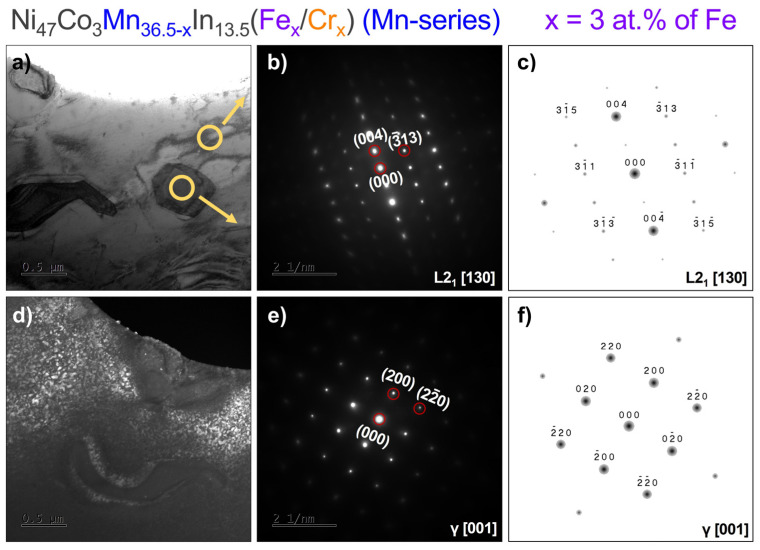
Bright- (**a**) and dark-field (**d**) TEM images with the corresponding Selected-Area Electron Diffraction Patterns (SAEDPs) from the matrix (**b**) and γ particle (**e**) in 3 at.% of Cr in the Mn-series and simulated electronograms for L2_1_ (**c**) and γ (**f**) phases in the proper zone axis.

Transmission electron microscopy observation was performed to determine the structure of the phases. The results for the Ni_47_Co_3_Mn_33.5_In_13.5_Fe_3_ alloy (x = 3 at.% of Fe in the Mn-series) are shown in Figure 6. The analysis of Selected-Area Electron Diffraction Patterns (SAEDPs) of the matrix and precipitates confirmed the ordered L2_1_ structure of austenite and the γ phase of particles. Figure 6d shows a dark-field (DF) image recorded from the L2_1_ spot. The white areas in the DF image correspond to the anti-phase domain of the long-range L2_1_ ordering. The dimensions of the anti-phase domain are approximately five times smaller than previously observed in quaternary alloys in bulk form after homogenization at 900 °C at 24 h and quenching (our work [32]). It may be an essential factor in microstructure modification in terms of the martensitic transformation arrest, which was observed in Ni-Co-Mn-In alloys and described in [33].

### 3.5. Differential Scanning Calorimetry (DSC)

**Figure 7:**
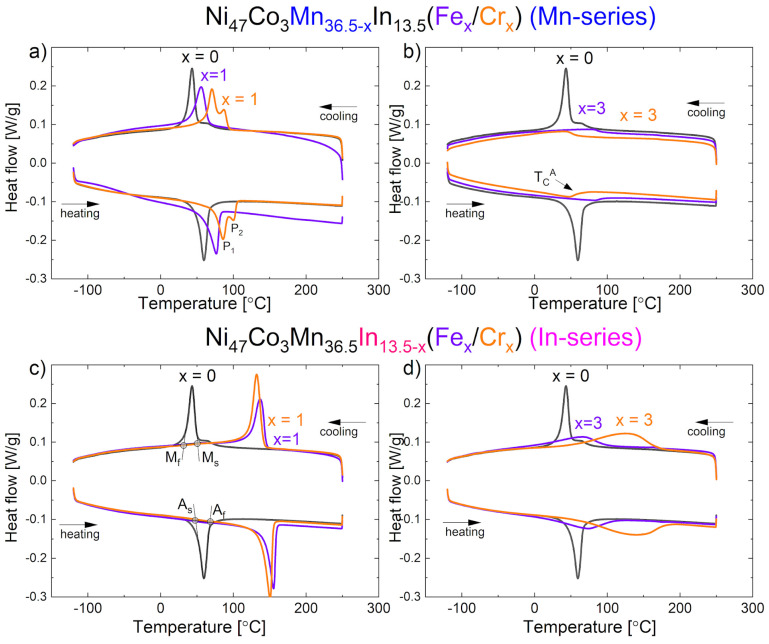
DSC heating/cooling curves for Ni_47_Co_3_Mn_36.5−x_In_13.5_ (denoted as Mn-series) for x = 0 and 1 at.% Cr/Fe (**a**) and x = 0 and 3 at.% Cr/Fe (**b**), and for Ni_47_Co_3_Mn_36.5_In_13.5−x_ (denoted as In-series) for x = 0 and 1 at.% Cr/Fe (**c**) and x = 0 and 3 at.% Cr/Fe (**d**).

The DSC measurements were carried out to predict the functional properties of alloys and determine the martensitic transformation behavior. The DSC heating/cooling curves for the Ni_47_Co_3_Mn_36.5−x_In_13.5_ (Mn-series) and Ni_47_Co_3_Mn_36.5_In_13.5−x_ (In-series) alloys for x = 0, 1, and 3 at.% Cr/Fe are presented in Figure 7. The thermal effect visible as a peak during cooling is related to martensitic transformation (forward) and that visible during heating is related to reverse MT. The heat flow was normalized to the sample size (divided by mass to exclude the sample mass effect). The MT temperatures, forward (M_s_—martensitic start; M_f_—martensitic finish; T_M_—peak temperature) and reverse (A_s_—austenitic start; A_f_—austenitic finish; T_A_ —peak temperature), with the enthalpy of transformation ∆H, temperature range (M_s_–M_f_ and A_f_–As), and hysteresis A_f_–M_s_, are shown in Table 3.

**Figure 8:**
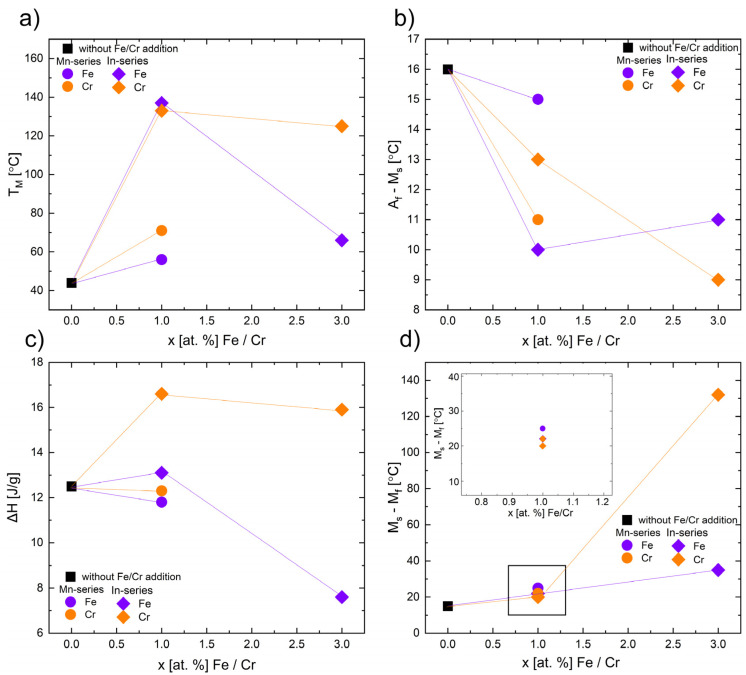
Effect of the addition of Fe (purple-colored) and Cr (orange-colored) on the martensitic transformation (MT) behavior in Ni_47_Co_3_Mn_36.5−x_In_13.5_ (Mn-series—diamond points) and Ni_47_Co_3_Mn_36.5_In_13.5−x_ (In-series—circle points), where x = 0 is marked as a square: temperature peak T_M_ (**a**), hysteresis A_f_–M_s_ (**b**), total enthalpy ∆H (**c**), and temperature range (M_s_–M_f_) (**d**). The inset in (**d**) shows a magnified view of the selected region of the M_s_–M_f_.

The quaternary alloy exhibits narrow peaks during cooling and heating with T_M_ = 44 °C and T_A_ = 58 °C. Addition of 1 at.% of Fe and 1 at.% of Cr influenced the martensitic transformation and caused the increase in the MT temperatures (M_s_, M_f_, T_M_, A_s_, A_f_, T_A_). The increase in MT temperature peak T_M_ was higher in the In-series (x = 1 at.% of Fe, T_M_ = 137 °C) than in the Mn-series and (x = 1 at.% of Fe, T_M_ = 56 °C) (Figure 7a,c, and Figure 8a). On the other hand, the peak shift was higher for the Cr-doped alloy than for the Fe-doped alloy in the Mn-series.

The addition of 1 at.% of Fe and 1 at.% of Cr in the In-series caused a shift in the T_M_ peak positions to 137 and 133 °C, respectively. In these alloys, the total enthalpy increased from 12.5 J/g (x = 0) to 13.1 J/g (x = 1 at.% of Fe) and 15.9 J/g (x = 1 at.% of Cr) in the In-series.

In the alloy with 1 at.% of Cr in the Mn-series, a two-step martensitic transformation (forward and reverse) was observed. This may be a result of some chemical composition segregation.

The samples with 3 at.% of Fe or Cr in the In-series exhibited a broadened martensitic transformation peak with an increase in peak position compared to the quaternary alloy.

**Figure 9:**
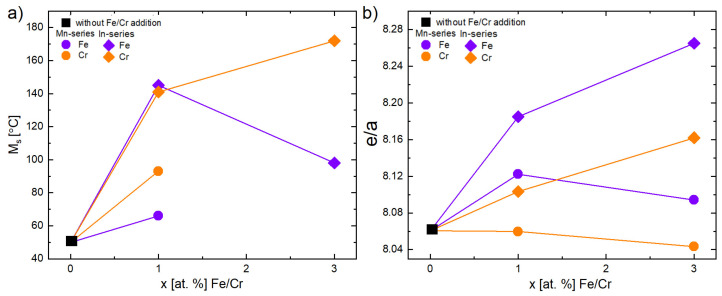
Martensitic transformation temperature M_s_ (**a**) and electron concentration ratio (e/a) (calculated based on EDS average composition) (**b**) depending on at.% Fe (purple) and at.% Cr (orange) in Ni_47_Co_3_Mn_36.5−x_In_13.5_ (Mn-series—diamond points) and Ni_47_Co_3_Mn_36.5_In_13.5−x_ (In-series—circle points).

The temperature range of martensitic transformation increased, especially for the Cr-doped alloy, which is clearly visible in the plot in Figure 9a. For the addition of 3 at.% of Fe and Cr in the Mn-series, the martensitic transformation was not observed. Some reports proved that the solid solution limit of Fe in Ni–Mn–In alloy is about 4 at.% [20]. Fang et al. [24] showed that the addition of 3 at.% of Fe caused the disappearance of the magnetic transition in austenite, and a martensitic transformation occurred between paramagnetic austenite and weak-magnetic martensite. The observed effect on DSC curves for 3 at.%-doped samples in the Mn-series (Figure 9b) is related to the magnetic transition of austenite (T_C_^A^). The XRD and EBSD structure investigations proved the presence of the austenite phase at room temperature. The Curie temperature of austenite (T_C_^A^) was also observed in the quaternary sample (x = 0). For other samples, it is possible that the Curie temperature is hidden by the peak from martensitic transformation. To clarify this issue, more investigations using various techniques are needed. Based on the DSC results, an increase in T_C_^A^ was observed in Fe-doped alloys, while a slight decrease was observed in Cr-doped alloys. The largest difference was observed in the sample with 3 at.% of Fe in the In-series (168 °C) compared to the quaternary alloy (T_C_^A^ = 68 °C).

The hysteresis of martensitic transformation (A_f_–M_s_) slightly decreased for each Fe- and Cr-doped alloy. A small hysteresis is desired in shape memory alloys [34]. However, the MT temperature range (M_s_–M_f_) slightly increased for 1 at.% of Fe and Cr samples and significantly increased for 3 at.% of Cr in the In-series (up to 132 °C). Based on these parameters, the alloy with 1 at.% of Fe in the Mn-series seems to be the most beneficial (hysteresis A_f_–M_s_ = 10 °C, MT temperature range M_s_–M_f_ = 22 °C; for comparison, the x = 0 alloy exhibited A_f_–M_s_ = 17 °C and M_s_–M_f_ = 25 °C), but only in application at higher temperatures (because T_M_ = 137 °C).

To summarize the effect of Fe and Cr addition on MT temperatures, it may be noticed that T_M_ (Figure 8a) increased with the addition of 1 at.% of Fe and Cr and then decreased with 3 at.% of Fe and Cr (in the In-series), while MT disappeared in the Mn-series. The enthalpy ∆H of transformation (Figure 8c) shows similar relationships.

Interestingly, predictions based on theoretically and experimentally calculated e/a (Table 1 and Table 2) show a decrease in e/a with an increase in at.% of Cr in the Ni_47_Co_3_Mn_36.5−x_In_13.5_ (Mn-series) alloys. It should be determined that the M_s_ temperature also decreases. However, the experimental results showed otherwise (Figure 9). A similar anomalous effect was observed in the Ni_2_Mn_1−x_Cr_x_Ga alloys [35]. The authors explained that the increase in M_s_ cannot be related only to e/a. Other factors like hybridization and electronegativity should be incorporated as well.

The predictions and experimental results are in agreement for 1 at.% Fe or Cr doped into the Ni_47_Co_3_Mn_36.5_In_13.5−x_ alloys (In-series) and 1 at.% Fe doped into the Ni_47_Co_3_Mn_36.5−x_In_13.5_ alloys (Mn-series). In the alloys with 3 at.% addition, the decrease in MT or change in the slope of the trend line is observed (Figure 9a). This change is correlated with the increase in γ volume fraction, which suppressed the martensitic transformation by introducing local stresses and decreasing the e/a of matrix (the measured e/a of γ is higher than that of the matrix and is about 8.7). The existence of the precipitates enhances the fracture strength of Ni–Ni-Mn-based alloys, but hinders the martensitic transformation. However, a proper amount and distribution of the γ phase may be beneficial for obtaining a good elastocaloric effect in Ni–Mn-based alloys [16].

According to the DSC results, the highest austenite volume is expected in 3 at.% Fe- or Cr-doped alloys in the Mn-series due to the lack of martensitic transformation peaks. However, the presence of residual martensite was observed in the 3 at.% Cr-doped alloy by EBSD (Figure 5e) and XRD (Figure 2b), which is not consistent with the DSC results. The occurrence of the residual martensite is probably related to other factors, like internal stresses and stress-induced martensite [36]. On the other hand, a lack of MT in 3 at.% Fe- or Cr-doped alloys may result from small dimensions of anti-phase domains (Figure 6d) [33]. In the Ni-Mn-Co-In alloy, the changes in Long-Range Atomic Order may affect the MT [37]. The increase in atomic order enhances the magnetic characteristic of the austenite, thus increasing its Curie temperature and lowering the MT temperature [38].

### 3.6. Microhardness

**Figure 10:**
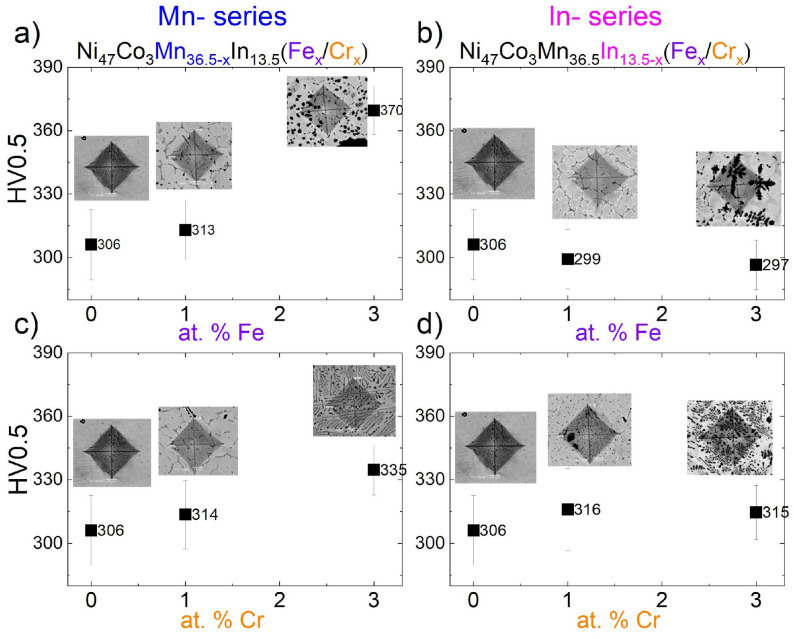
The average Vickers microhardness (HV0.5) depending on Fe (at the top) or Cr (at the bottom) contents in Ni_47_Co_3_Mn_36.5−x_In_13.5_ (Mn-series) (**a**,**b**) and Ni_47_Co_3_Mn_36.5_In_13.5−x_ (In-series) (**c**,**d**). Inset shows the SEM BSE images of a representative example of the imprint among the microstructure components.

The Vickers microhardness measurements were carried out to study the effect of Fe and Cr addition on the mechanical properties of Ni-Co-Mn-In alloys. Table 4 shows the average microhardness values with the standard deviation. Microhardness was measured at 60 points on the cross-section of each specimen. Figure 10 illustrates the relationship between average microhardness and the Fe or Cr content in Ni_47_Co_3_Mn_36.5−x_In_13.5_ (Mn-series) (a, b) and Ni_47_Co_3_Mn_36.5_In_13.5−x_ (In-series) for x = 0, 1, and 3 atomic percent (at.%) of Fe or Cr. The Vickers imprints were examined using SEM in BSE composition contrast mode to identify the microstructure elements (phases) present in each measurement (Figure 10). As one can see, microhardness increases with Fe addition in the Mn-series alloys and slightly decreases in the In-series. By contrast, microhardness increases with the Cr addition in both series.

A noticeable increase in microhardness was observed for the 3 at.% Fe-doped alloy (370(11) HV0.5) and for the 3 at.% Cr-doped alloy (335(12) HV0.5) in the Mn-series. For comparison, the quaternary alloy exhibited 306(17) HV0.5.

In the Mn-series, there are small particles along the grain boundaries and inside the grain, which is noticeable in the SEM images (Figure 3) and confirmed in the EBSD map (Figure 5c). In this series, the increase in hardness is correlated with microstructure refinement and a dispersion particle strengthening mechanism. The γ% fraction increased with an increasing amount of Fe and Cr (Table 4). The γ-phase may form a coherent interface with the matrix and nucleate at grain boundaries, which hinders intergranular fracture during cyclic loading [14].

Another trend in the evolution of microhardness is observed in the In-series alloys. A change in the morphology of the γ-phase from equiaxed to a dendritic-like shape is evident in alloys containing 3 at.% Fe or Cr in the In-series. Guo et al. [39] proved that the mechanical properties, i.e., compressive fracture strength and ductility, decreased with increasing γ grain size. This γ grain coarsening affects the dominant toughening mechanism, shifting it from crack-bridging to crack-deflection behavior [39]. Furthermore, the mechanical properties and cracking mechanism may also be influenced by elemental segregation at grain boundaries, where intermediate levels of chemical heterogeneity promote the highest crack propagation rates due to enhanced grain boundary decohesion [40].

In addition, it is possible that variations in the In concentration may determine changes in the hardness of alloys. Indium has the largest atomic radius (167 pm) compared to the substituted elements Fe (124 pm) and Cr (125 pm). Consequently, partial substitution of Indium by Fe or Cr (which reduces the In amount in the alloys) may suppress the precipitation strengthening effect. Enhanced microhardness after In addition was observed in Mg-Zn-based alloys. This was assigned to a solid strengthening mechanism and grain refinement of the alloy [41].

## 4. Conclusions

The addition of Fe or Cr elements to Ni_47_Co_3_Mn_36.5−x_In_13.5_(Fe_x_/Cr_x_) (Mn-series) and Ni_47_Co_3_Mn_36.5_In_13.5−x_(Fe_x_/Cr_x_) (In-series) caused the following:

1. Changes in the microstructure and phase composition:

- The Ni_47_Co_3_Mn_36.5−x_In_13.5_ quaternary alloy exhibits a coarse-grained martensitic structure (monoclinic, P2/m), while 1% Fe- and Cr-doped alloys exhibit a mixture of austenite (L2_1_, *Fm*$\bar{3}$*m*), martensite (monoclinic, P2/m), and γ particles enriched in Co and Fe/Cr, respectively, with a specific distribution in the matrix.
- The addition of 1% Fe caused higher microstructure refinement compared to the 1% Cr addition.
- Increasing the addition to 3 at.% caused an increase in the volume fraction of γ particles and their distribution, as well as changed their morphology (dendritic-like shape).

2. Changes in the martensitic transformation (MT) behavior:

- An increase in martensitic transformation (MT) temperatures was observed, which was higher in the In-series than in the Mn-series.
- This weakened or arrested the martensitic transformation in alloys containing additions of 3 at.% Fe or 3 at.% Cr, resulting in a total of 6 at.% of alloying elements when the 3 at.% Co already present in the alloy was included.

3. Changes in microhardness:

- An increase in microhardness from 306 HV0.5 (x = 0) to 370 HV0.5 (x = 3 at.% of Fe) and 335 HV0.5 (x = 3 at.% of Cr) in the Ni_47_Co_3_Mn_36.5−x_In_13.5_ (Fe_x_/Cr_x_) series was observed.
- The microhardness in the In-series and in the 1 at.%-doped (Fe/Cr) Mn-series remained at a similar level, irrespective of the morphology and γ particle size.

## Figures and Tables

**Table 1:** Nominal compositions of Ni_47_Co_3_Mn_36.5−x_In_13.5_ (x = 1, 3 at.% Cr/Fe) alloys (denoted as Mn-series) and Ni_47_Co_3_Mn_36.5_In_13.5−x_ (x = 1, 3 at.% Cr/Fe) alloys (denoted as In-series) with the electron concentration ratio (e/a). The arrows in the e/a column indicate its increase or decrease in a given series in relation to the quaternary alloy.

	Ni	Mn	Co	In	Fe	Cr	e/a	Short Name
	**47.0**	**36.5**	**3.0**	**13.5**	**-**	**-**	**7.93**	**x = 0**
**Mn-series**	47.0	35.5	3.0	13.5	1.0	-	7.94 ↑	x = 1
	47.0	33.5	3.0	13.5	3.0	-	7.96 ↑	x = 3
	47.0	35.5	3.0	13.5	-	1.0	7.92 ↓	x = 1
	47.0	33.5	3.0	13.5	-	3.0	7.90 ↓	x = 3
**In-series**	47.0	36.5	3.0	12.5	1.0	-	7.98 ↑	x = 1
	47.0	36.5	3.0	10.5	3.0	-	8.08 ↑	x = 3
	47.0	36.5	3.0	12.5	-	1.0	7.96 ↑	x = 1
	47.0	36.5	3.0	10.5	-	3.0	8.02 ↑	x = 3

**Table 2:** EDS average composition of Ni_47_Co_3_Mn_36.5−x_In_13.5_ (x = 1, 3 at.% Cr/Fe) alloys (denoted as Mn-series) and Ni_47_Co_3_Mn_36.5_In_13.5−x_ (x = 1, 3 at.% Cr/Fe) alloys (denoted as In-series) with the electron concentration ratio (e/a).

	Ni	Mn	Co	In	Fe	Cr	e/a	Short Name
**Mn-series**	**51.1**	**34.0**	**2.1**	**12.8**	**0.0**	**0.0**	**8.06**	**x = 0**
	52.2	33.2	1.7	12.1	0.8	0.0	8.12	x = 1
	51.3	31.5	1.9	12.7	2.6	0.0	8.09	x = 3
	51.3	33.8	1.7	12.7	0.0	0.5	8.06	x = 1
	50.7	32.3	2.3	12.5	0.0	2.3	8.04	x = 3
**In-series**	52.6	33.9	2.0	11.0	0.6	0.0	8.18	x = 1
	51.9	34.0	2.2	9.1	2.8	0.0	8.26	x = 3
	51.8	33.9	1.8	12.1	0.0	0.3	8.10	x = 1
	51.1	34.8	2.0	9.8	0.0	2.3	8.16	x = 3

**Table 3:** The martensitic transformation (MT) temperatures of forward (M_s_—martensitic start; M_f_—martensitic finish; T_M_—peak temperature) and reverse (A_s_—austenitic start; A_f_—austenitic finish; T_A_—peak temperature) with the Curie temperature of austenite (T_C_^A^), the enthalpy of transformation ∆H, the temperature range (M_s_–M_f_ and A_f_–A_s_), and hysteresis A_f_–M_s_ for Ni_47_Co_3_Mn_36.5−x_In_13.5_ (Mn-series) and Ni_47_Co_3_Mn_36.5_In_13.5−x_ (In-series) for x = 0, 1, and 3 at.% of Fe (purple) and Cr (orange).

	M_s_ [°C]	T_M_ [°C]	M_f_ [°C]	M_s–_M_f_	ΔH [J/g]	T_C_^A^ [°C]	A_s_ [°C]	T_A_ [°C]	A_f_ [°C]	A_f_–A_s_ [°C]	A_f_–M_s_ [°C]	ΔH [J/g]
**x = 0**	**50**	**44**	**35**	**15**	**−11.2**	**68**	**49**	**58**	**66**	**17**	**16**	**12.5**
**Ni_47_Co_3_Mn_36.5−x_In_13.5_(Fe_x_/Cr_x_)** **(Mn-series)**												
**x = 1**	66	56	41	25	−10.4	-	59	75	81	22	15	11.8
**x = 1**	93	P_1_ 71 P_2_ 88	61	22	−12.3	-	72	P_1_ 85 P_2_ 100	104	32	11	12.3
**x = 3**	-	-	-		-	87	-	-	-			-
**x = 3**	-	-	-		-	51	-	-	-			-
**Ni_47_Co_3_Mn_36.5_In_13.5−x_(Fe_x_/Cr_x_)** **(In-series)**												
**x = 1**	145	137	123	22	−12.5	-	142	154	158	16	10	13.1
**x = 1**	141	133	121	20	−17.1	-	137	149	154	17	13	16.6
**x = 3**	98	66	7	35	−7.9	168	10	73	109	99	11	7.6
**x = 3**	172	125	40	132	−20.4	-	60	134	181	121	9	15.9

**Table 4:** The average microhardness (HV0.5) for Ni_47_Co_3_Mn_36.5−x_In_13.5_ (Mn-series) and Ni_47_Co_3_Mn_36.5_In_13.5−x_ (In-series) for x = 0, 1, and 3 at.% of Fe or Cr with an indication of the phase composition: M—martensite; A—austenite; γ-phase precipitates.

	at.%	Mn-Series	Phase	γ Fraction	In-Series	Phase	γ Fraction
at.% Fe	x = 0	306 (17)	M	0%	306 (17)	M	0%
	x = 1	313 (14)	M + A + γ	5.0%	299 (14)	M + A + γ	7.0%
	x = 3	370 (11)	A + γ	6.4%	297 (12)	M + A + γ	12.9%
at.% Cr	x = 0	306 (17)	M	0%	306 (17)	M + A + γ	0%
	x = 1	314 (16)	M + A + γ	6.7%	316 (19)	M + A + γ	6.8%
	x = 3	335 (12)	M + A + γ	15.4%	315 (13)	M + A + γ	19%

## Data Availability

The original contributions presented in this study are included in the article. Further inquiries can be directed to the corresponding author.

## Abbreviations

The following abbreviations are used in this manuscript:

BSE	Backscattered Electron
DSC	Differential Scanning Calorimetry
EBSD	Electron Backscatter Diffraction
EDM	Electric Discharge Machine
EDS	Energy-Dispersive X-ray Spectroscopy
FSMA	Ferromagnetic Shape Memory Alloy
MCE	Magnetocaloric Effect
MT	Martensitic Transformation
RC	Refrigeration Capacity
SEM	Scanning Electron Microscopy
TEM	Transmission Electron Microscopy
VAR	Vacuum Arc Melting
XRD	X-Ray Diffraction
